# Reducing Compaction Temperature of Asphalt Mixtures by GNP Modification and Aggregate Packing Optimization

**DOI:** 10.3390/ma15176060

**Published:** 2022-09-01

**Authors:** Tianhao Yan, Mugurel Turos, Jia-Liang Le, Mihai Marasteanu

**Affiliations:** Department of Civil, Environmental and Geo- Engineering, University of Minnesota, Twin Cities, Minneapolis, MN 55455, USA

**Keywords:** temperature reduction, compaction, asphalt mixture, Graphite Nanoplatelets (GNP), aggregate packing

## Abstract

Compaction of hot mix asphalt (HMA) requires high temperatures in the range of 125 to 145 ${}^{{}^{\circ}}$C to ensure the fluidity of asphalt binder and, therefore, the workability of asphalt mixtures. The high temperatures are associated with high energy consumption, and higher NOx emissions, and can also accelerate the aging of asphalt binders. In previous research, the authors have developed two approaches for improving the compactability of asphalt mixtures: (1) addition of Graphite Nanoplatelets (GNPs), and (2) optimizing aggregate packing. This research explores the effects of these two approaches, and the combination of them, on reducing compaction temperatures while the production temperature is kept at the traditional levels. A reduction in compaction temperatures is desired for prolonging the paving window, extending the hauling distance, reducing the energy consumption for reheating, and for reducing the number of repairs and their negative environmental and safety effects, by improving the durability of the mixtures. A Superpave asphalt mixture was chosen as the control mixture. Three modified mixtures were designed, respectively, by (1) adding 6% GNP by the weight of binder, (2) optimizing aggregate packing, and (3) combining the two previous approaches. Gyratory compaction tests were performed on the four mixtures at two compaction temperatures: 135 ${}^{{}^{\circ}}$C (the compaction temperature of the control mixture) and 95 ${}^{{}^{\circ}}$C. A method was proposed based on the gyratory compaction to estimate the compaction temperature of the mixtures. The results show that all the three methods increase the compactability of mixtures and thus significantly reduce the compaction temperatures. Method 3 (combining GNP modification and aggregate packing optimization) has the most significant effect, followed by method 1 (GNP modification), and method 2 (aggregate packing optimization).

## 1. Introduction

High temperature is required for the construction of hot mix asphalt (HMA), which is typically 145 to 165 ${}^{{}^{\circ}}$C for producing (mixing) asphalt mixtures, and 125 to 145 ${}^{{}^{\circ}}$C for paving and compacting. The high temperatures lower the viscosity of asphalt binders and thus ensure the workability of asphalt mixtures [1,2]. However, high production temperature increases the fuel consumption for heating, the emission of greenhouse gases and pollution gases, and the aging of asphalt binders. High compaction temperature shortens the hauling distance, the time window for compaction, and the paving season every year [3].

The most widely used temperature reduction technology is the Warm Mix Asphalt (WMA), which reduces the production and compaction temperatures of HMA by 20–40 ${}^{{}^{\circ}}$C [3,4]. The temperature reduction of WMA is typically achieved by one of the following approaches: (1) the addition of organic additives, (2) the addition of chemical additives, or (3) the foaming processes [5,6]. Organic additives are mainly waxes. They melt below the melting point of the binder, thus reducing the viscosity of binder during mixing and compaction which improves the coating and workability of the mix [4]. Chemical additives are in the form of emulsions and surfactants, which work at the microscopic interface of the binder and aggregates to reduce the frictional forces and facilitate lubrication between the binder and aggregate during mixing and compaction [7,8]. Foaming technologies involve the introduction of small amounts of water into the asphalt binder. The water evaporates during the mixing process with the hot binder and, therefore, the steam remains entrapped, generating a large volume of foam. The foam causes a temporary viscosity reduction in the mix which results in improved aggregate coating and easier compaction of the asphalt mix at lower temperatures [5,9]. Numerous studies have shown the benefits of WMA on mitigating the issues caused by the high mixing and compaction temperatures [10,11,12,13,14,15,16].

In previous studies, the authors have developed two different approaches for improving the compactability of asphalt mixtures, which are (1) the addition of Graphite Nanoplatelets (GNPs) [17,18,19,20] and (2) the optimization of aggregate packing [21]. In these studies, the authors demonstrated that both methods increase the compactability of asphalt mixtures, while retaining or improving relevant mechanical properties of asphalt mixtures, such as resistance to rutting, cracking, and fatigue [18,21]. Given the improvement in the compactability of asphalt mixtures, it is expected that these two methods can reduce the compaction temperature of asphalt mixtures.

The objective of this study is to explore the effects of the two methods (adding GNP and optimizing aggregate packing) and the combination of them on reducing compaction temperatures. It is important to note that, although the GNP modification could also lead to a reduction of the mixing temperature, the focus of this study is only on the reduction of compaction temperature. Therefore, the mixing temperature is maintained at the traditional level in this study. The produced mixtures are therefore in between HMA and WMA, which can be classified as the hot-mixed warm-compacted (HMWC) mixtures, meaning that the mixtures are mixed at the same temperature as HMA but compacted at a lower temperature than HMA [22]. Similar to WMA, the HMWC mixtures have shown many advantages over HMA due to the reduction of compaction temperature, for example, (1) extending the hauling distance (beneficial for paving at remote locations and emergency paving after natural disasters) [22,23], (2) facilitating cold-weather paving [24], (3) extending the paving window [25], and (4) reducing the energy consumption for reheating. Moreover, in some cases, the mixing temperatures of mixtures are predominantly governed by the compaction temperature and the required hauling distance and paving window [26]. Under such circumstances, the reduction of compaction temperature helps reducing the mixing temperature as well.

In addition, a new method is proposed to determine the compaction temperature. Most of previous methods assume that compaction is governed by the viscosity of asphalt binder and therefore determine the compaction temperature by the viscosity–temperature relationship of asphalt binder [27,28]. For polymer-modified binders (typically non-Newtonian), the viscosity is shear rate-dependent, so it has been argued that viscosity should be measured at the shear rate representative for compaction of asphalt mixtures. However, the representative shear rate is still under debate [1,2,27,28,29,30]. These methods use the binder viscosity measured by a Rotational Viscometer (RV). The Dynamic Shear Rheometer (DSR) has also been used for measuring the binder viscosity and determining the compaction temperature [31]. In addition to the viscosity-based methods, phase angle of asphalt binder (measured by DSR) was also used as an index for determining compaction temperature [32]; it is assumed that the change in phase angle characterizes the solid to liquid transition of asphalt binder. It is important to note that all these methods are based on rheological properties (viscosity or phase angle) of asphalt binder. However, many studies have questioned this approach, since the compactability of asphalt mixtures is also dependent on many other factors, such as binder content, lubrication of binder, aggregate gradation, and angularity [33,34,35].

In this study, a new method for determining the compaction temperature is proposed based on characterizing the compactability of mixtures by the gyratory compaction. The density at 30 gyrations ($\phi_{30}$) is selected as compactability index, since previous studies have shown that the $\phi_{30}$ approximately represents the field density of the mixture [36,37]. Given the desired field density, the compaction temperature is interpolated by gyratory compaction results at different temperatures.

The paper is structured as follows. First, the two methods for improving compactability are introduced in Section 2. An experimental study is then performed to investigate the effects of the two methods and their combination on reducing the compaction temperature. The material information and experimental plan of gyratory compaction are detailed in Section 3, and the results of gyratory compaction and the method for estimating compaction temperatures are presented and discussed in Section 4.

## 2. Two Approaches for Improving Compactability

### 2.1. GNP Modification

The GNPs are nano-discs with a sub-micrometer diameter and a thickness on the order of nanometer. The GNPs could be produced from either graphene or natural graphite. If the GNPs are prepared directly from graphene, each platelet typically consists of several layers of graphene sheets, each of which is a single layer of carbon atoms. Depending on its type and carbon purity, the cost of GNPs can be as low as $3 per pound, which is comparable to some existing asphalt modifiers such as the SBS.

Recent studies have shown that addition of small amount (3% to 6% by weight of the binder) of GNPs to asphalt binder can result in 20% to 40% reduction in the compaction effort required for achieving the target air voids, 130% increase in the flexural strength of asphalt binders, and almost 100% increase in the fracture energy of asphalt mixtures. Meanwhile, other mechanical properties of binders and mixtures, e.g., rutting, stiffness, fatigue, and rheology, are not adversely affected by the GNP modification [17,18].

**Figure 1:**
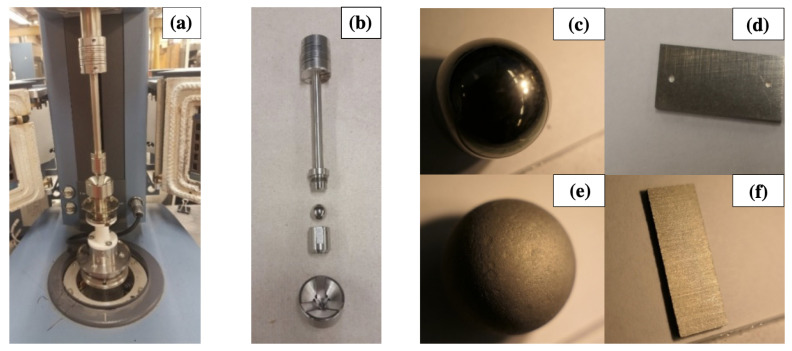
Tribological test fixture: (**a**) general view, (**b**) components of the fixture, (**c**) smooth ball, (**d**) smooth plate (used), (**e**) rough ball, (**f**) rough plate.

The physical mechanisms of GNPs on improving the compactability of asphalt mixtures have been investigated in the past years [19,20]. It was found that the GNP addition increases the viscosity of the binder, which implies that the improved compactability cannot be attributed to the viscosity of asphalt binder. A tribological test was developed measuring the lubricating effect of binder between rough surfaces [19]. The test fixture is shown in Figure 1. As shown in Figure 1a, the tribological test fixture is developed based on the AR 2000 TA Dynamic Shear Rheometer (DSR) equipment (TA Instruments, New Castle DE, USA). As shown in Figure 1b, the fixture contains five different components: a lower cup, three steel plates, a steel ball, a shaft, and a ring to attach the ball to the shaft. In the lower cup, there are three plates with an angle of 45${}^{{}^{\circ}}$ with respect to the horizontal plane and the asphalt sample. The steel ball is attached to the shaft, which then gets attached to the DSR head. To better simulate the surface of the aggregates, the surfaces of the ball and the plates were roughened by immersing the ball and the plates in hydrochloric acid (HCl) for a period of time. Hydrochloric acid corroded the surfaces of the parts and made them rough and looking like an orange skin. The roughness increases with the corroding period. It was found that a corroding period of three days best simulates the roughness of aggregate surfaces. Therefore, the corroding period was strictly controlled at three days, to achieve consistent roughness for all the testing balls and plates. Figure 1c–f compares the original smooth ball and plate and the ball and plate after they were roughened by the acid. During the tribological test, the axial force was kept constant and equal to 10 N, whereas the rotational speed was increased in logarithmic steps from 0.1 to 1433 rpm. The friction coefficient was measured as the function of the rotational speed. The results showed that the addition of GNPs lowers the friction coefficient between rough surfaces and therefore explained the effect of GNPs on improving the compactability of mixtures.

**Figure 2:**
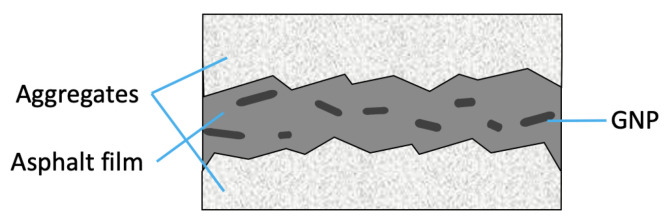
Scheme of the GNP-modified asphalt film between aggregate surfaces.

Microscopically, it is believed that GNPs occupy the space between the asperities of the aggregates as shown in Figure 2, which improve the lubricating effect of asphalt binders and, thus, the compactability of asphalt mixtures.

### 2.2. Aggregate Packing Optimization

In a recent study on developing the Superpave 5 asphalt mix designs in Minnesota [21], a method was developed to improve the compactability of asphalt mixtures by optimizing the aggregate packing. In this method, aggregate sources are divided into the coarse and fine aggregates. Then, the mass ratio between the coarse and fine aggregates (C/F ratio) is adjusted to maximize the packing density of the mixture. This method is based on the binary aggregate packing theory which shows that when mixing two aggregate sources of different sizes, there exists an optimum C/F ratio which raises the maximum packing density for the mixture [38,39,40,41,42,43,44].

**Figure 3:**
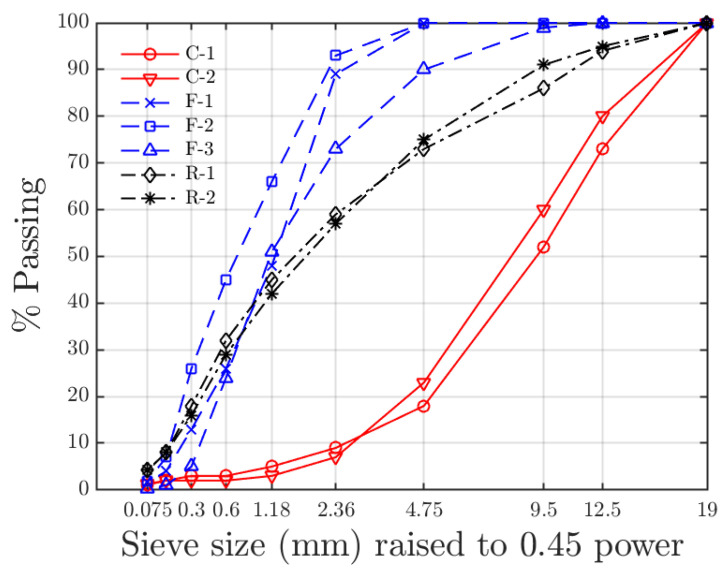
Gradation of aggregate sources. The line colors and styles indicate the type of aggregate.

One mixture from a previous study [21] is used to demonstrate this mix design method. The mixture was originally designed as a traditional Superpave mixture (4% air voids at N_design_ = 60) and was modified to a Superpave 5 mixture (5% air voids at N_design_ = 30) by this method. The mixture contains five aggregate sources and two reclaimed asphalt pavements (RAP). The aggregate gradations of the aggregate sources are plotted in Figure 3. As shown, aggregate sources are separated into the coarse aggregates (C-1 and C-2) and fine aggregates (F-1, F-2, and F-3) and RAP’s (R-1 and R-2).

**Figure 4:**
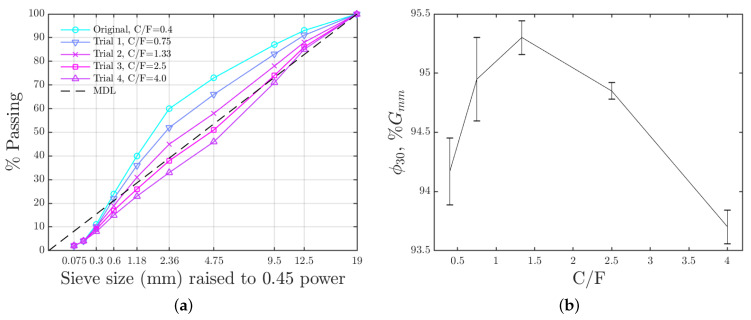
(**a**) Aggregate gradations of the trial blends. (**b**) Densities of the trial blends at 30 gyrations in gyratory compaction. The error bar represents the difference between the two replicates.

Trial blends were designed by adjusting the C/F ratio of the aggregates while keeping the binder content (5.6%) and the RAP contents (20% for R-1 and 10% for R-2) unchanged. The aggregate gradations of the Trial blends are shown in Figure 4a. It is seen that as the C/F ratio increases, the aggregate gradation changes from fine-graded to coarse-graded. Densities of mixtures (ϕ) at 30 gyrations, $\phi_{30}$, are plotted in Figure 4b. It is seen that the Trial 2 (C/F = 1.33) has the densest aggregate packing and thus achieves the maximum $\phi_{30}$, 95.25 %G_mm_, which represents a 1.08% increase compared to the original mix design. Therefore, the aggregate gradation of the Trial 2 (C/F = 1.33) was selected for the modified mix design.

**Figure 5:**
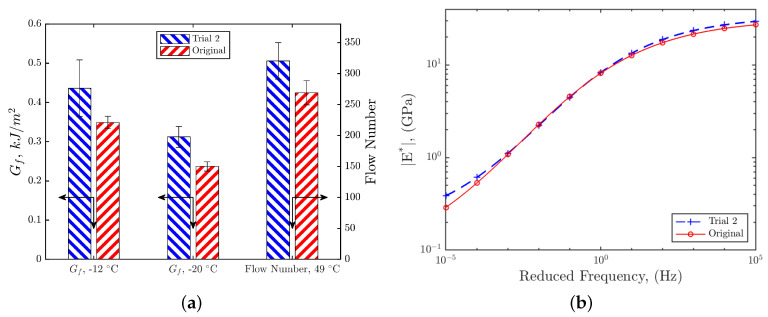
Performance tests results of the original and modified mixtures. (**a**) SCB fracture energy and flow number results. The error bar indicates the standard error of three replicates. The arrows indicate the corresponding axes for the data. (**b**) Dynamic modulus master curves where the reference temperature is 12 ${}^{{}^{\circ}}$C.

Laboratory performance tests were conducted to compare the mechanical properties of the modified and the original mixtures. They are the Semi-Circular Bending (SCB) [45], Flow Number [46], and Diametral dynamic modulus [47]. Test specimens are prepared by gyratory compaction to 30 gyrations. The average densities of the modified mixture and the original mixture are 94.17 %G_mm_ and 95.25 %G_mm_, respectively. The performance test results are shown in Figure 5. It is seen that the modified mixture (Trial 2) had higher fracture energy (at both −12 and −20 ${}^{{}^{\circ}}$C), flow number, and dynamic modulus than the original mixture, indicating that the modified mixture would have better performance in the field than the original mixture.

## 3. Experimental Study

### 3.1. Materials Information

The traditional Superpave asphalt mixture mentioned in Section 2.2 was used as the control mixture (Mix-C) in this study. Mix-C was used for the wearing course of a traffic level 3 (20-year design Equivalent Single Axle Loads (ESAL’s) are between 1 to 3 million) project in Minnesota in 2020. It was designed at 4% air voids with the N_design_ = 60. The aggregate gradation of the Mix-C is shown in Figure 4a as the original gradation. More information of the Mix-C is listed in Table 1.

Based on the raw materials of the Mix-C, three modified mixtures were designed by (1) GNP modification, (2) optimizing aggregate packing, and (3) a combination of GNP modification and optimizing aggregate packing. The first modified mixture is denoted as Mix-GNP. It modified Mix-C by adding 6% GNPs by the weight of binder to the mixture. The GNPs used in this study are made of a synthetic graphite material with 99.66% carbon and 0.34% ash, characterized by an enhanced surface area equal to 250 m^2^/g. The same GNPs have been used in previous studies [17,18,19,20]. In this study, the GNPs were added into the mixture during the mixing process after aggregates and binder were thoroughly mixed for 5 minutes by a mechanical mixer at 145 ${}^{{}^{\circ}}$C.

The second modified mixture was designed by optimizing aggregate packing. Details of this approach is described in Section 2.2. This modified mixture is denoted as Mix-Agg. The aggregate gradation of Mix-Agg is shown in Figure 3a as that of the Trial 2. Other properties of Mix-Agg are the same as Mix-C as listed in Table 1.

The third modified mixture was designed by combining the two former approaches. It contains 6% GNPs while it has the optimized aggregate gradation of the Mix-Agg. This modified mixture is denoted as Mix-GNP&Agg.

### 3.2. Experimental Plan

Laboratory gyratory compaction tests (AASHTO T312) [48] were performed for the four mixtures to 100 gyrations at two compaction temperatures: 135 ${}^{{}^{\circ}}$C and 95 ${}^{{}^{\circ}}$C. The 135 ${}^{{}^{\circ}}$C was chosen since it is the recommended compaction temperature for Mix-C. Since the focus of this study is on the reduction of compaction temperature, the mixing temperature was kept constant at 145 ${}^{{}^{\circ}}$C for all mixtures. For each mixture, two samples were compacted. Based on the gyratory compaction results at the two reference temperatures, the compaction temperatures of the modified mixtures are estimated by the direct characterization of the compactability of mixtures by gyratory compaction.

## 4. Results and Discussion

### 4.1. Gyratory Compaction Results

**Figure 6:**
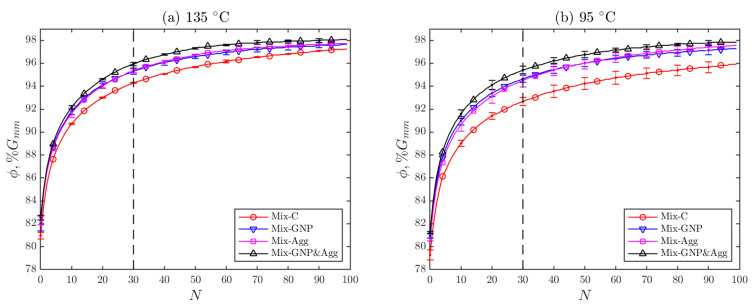
Gyratory compaction results at (**a**) 135 ${}^{{}^{\circ}}$C and (**b**) 95 ${}^{{}^{\circ}}$C.

The gyratory compaction curves of the four mixtures at the two compaction temperatures (135 ${}^{{}^{\circ}}$C and 95 ${}^{{}^{\circ}}$C) are shown in Figure 6. The compaction curves show the average of two replicates, while the error bars show the differences between the two replicates. It is seen that the three modified mixtures have higher compactability than the control mixture (Mix-C) at both compaction temperatures. Among the modified mixtures, Mix-GNP&Agg is the most compactable, followed by Mix-GNP and Mix-Agg. The compaction curves of Mix-GNP and Mix-Agg are very close to each other at both temperatures, but it is seen that Mix-GNP is slightly more compactable than Mix-Agg at the starting phase of gyratory compaction while Mix-GNP is surpassed by Mix-Agg at the later phase of gyratory compaction. It is also noticed that the variability of compaction curves is higher at 95 ${}^{{}^{\circ}}$C than at 135 ${}^{{}^{\circ}}$C, which indicates that lower compaction temperature may increase the variability of field density.

**Figure 7:**
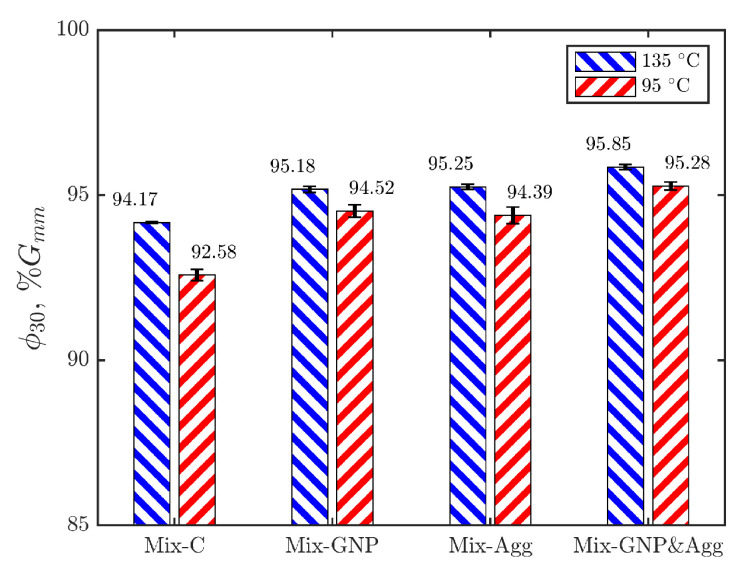
Bar chart of $\phi_{30}$ at the two compaction temperatures.

Previous studies have shown that the field compaction effort of current asphalt pavement projects is approximately equivalent to 30 gyrations in the gyratory compaction [36,37]. Therefore, we use the density of mixtures at 30 gyrations, $\phi_{30}$, to characterize the compactability of the mixture. The higher the $\phi_{30}$, the more compactable the mixture is. The results of $\phi_{30}$ for the four mixtures at the two temperatures are shown in Figure 7, where the heights of the bars show the average $\phi_{30}$ of the two replicates, and the error bars show the difference of $\phi_{30}$ between the two replicates.

As shown in Figure 7, as expected, it is seen that mixtures are more compactable at 135 ${}^{{}^{\circ}}$C than at 95 ${}^{{}^{\circ}}$C, which can be attributed to the lower viscosity of binder at higher temperatures. The comparison between $\phi_{30}$ at 135 ${}^{{}^{\circ}}$C and 95 ${}^{{}^{\circ}}$C shows that the effect of temperature on compactability is more significant for the control mixture (Mix-C) than the modified mixtures. Moreover, it is seen again that the variability of compactability is higher at the lower compaction temperature.

**Figure 8:**
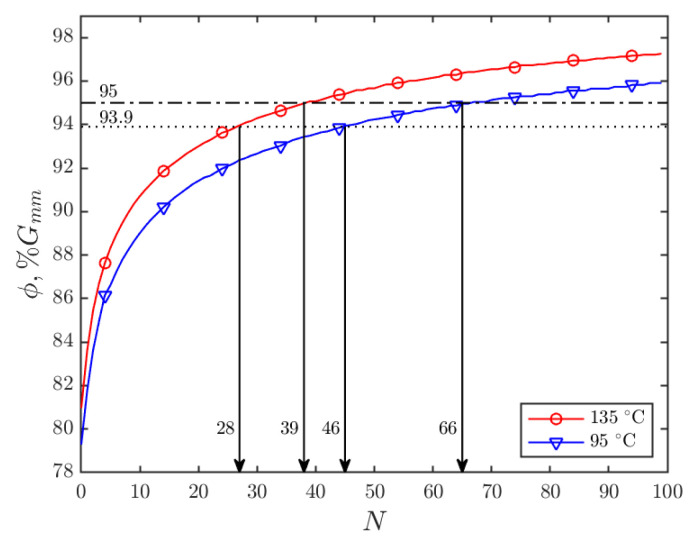
Calculation of the number of gyrations needed for achieving the two density levels for Mix-C.

**Figure 9:**
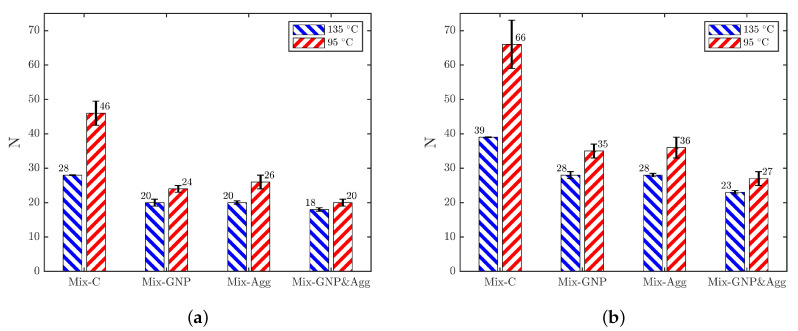
The numbers of gyrations needed for achieving the two density levels: (**a**) 93.9 %G_mm_ and (**b**) 95 %G_mm_.

Another way to evaluate the compactability of mixtures is by the corresponding number of gyrations needed for achieving a certain density level. In this study, two density levels are of particular interest: (1) the average field density of the control mixture (Mix-C) and (2) the required field density level of the Superpave 5 mix design. The average field density of the Mix-C was 93.9 %G_mm_, which was obtained from the quality control and quality assurance (QC/QA) data of the project. The required field density level of Superpave 5 mixtures is 95 %G_mm_, which is about 1–2% increase compared with the traditional Superpave mixtures [21,49]. Based on the gyratory compaction results, the numbers of gyrations needed for achieving the two density levels were calculated. An example of such procedure is shown in Figure 8 for Mix-C. The results of all mixtures are shown in Figure 9, where the heights of the bars show the average equivalent number of gyrations of the two replicates, and the error bars show the difference of equivalent number of gyrations between the two replicates.

As shown in Figure 9a , for achieving the average field density (93.9 %G_mm_), Mix-C needs 28 gyrations at the recommended compaction temperature (135 ${}^{{}^{\circ}}$C), which is consistent with previous studies [36,37], showing that the field compaction effort is approximately equivalent to 30 gyrations in gyratory compaction. Because of the improved compactability, the modified mixtures achieved 93.9 %G_mm_ at much lower compaction effort at 135 ${}^{{}^{\circ}}$C, which are 20, 20, and 18 gyrations for Mix-GNP, Mix-Agg, and Mix-GNP&Agg, respectively. At the lower compaction temperature (95 ${}^{{}^{\circ}}$C), due to the increase in binder viscosity, more compaction effort is needed than at 135 ${}^{{}^{\circ}}$C. The increase in number of gyrations is the most significant for Mix-C, which increased by 64% (from 28 to 46), while for the modified mixtures, such increase becomes less significant. It is important to note that, even at the lower compaction temperature 95 ${}^{{}^{\circ}}$C, all the modified mixtures need less compaction effort (25, 26, and 20 gyrations for Mix-GNP, Mix-Agg, and Mix-GNP&Agg, respectively) than the original compaction effort of Mix-C at 135 ${}^{{}^{\circ}}$C (28 gyrations), which implies that the improved compactability of the modified mixtures would enable them to be compacted at lower temperatures than 95 ${}^{{}^{\circ}}$C for achieving the density level of 93.9 %G_mm_. More details about the estimation of compaction temperatures will be discussed in Section 4.2.

Figure 9b shows the numbers of gyrations needed for achieving 95 %G_mm_, the density required by the Superpave 5 mix design. Similarly, it is seen that more compaction effort is needed at 95 ${}^{{}^{\circ}}$C than at 135 ${}^{{}^{\circ}}$C. The comparison between Figure 9a,b shows that, as expected, more compaction effort is needed for achieving 95 %G_mm_ than 93.9 %G_mm_. The increase is quite consistent, which is about 30% to 40% for all mixtures at both compaction temperatures. It is important to note that, at 135 ${}^{{}^{\circ}}$C, all the modified mixtures require less or equal compaction effort (28, 28, and 23 gyrations for Mix-GNP, Mix-Agg, and Mix-GNP&Agg, respectively) to achieving 95 %G_mm_ than the original compaction effort of Mix-C (28 gyrations). This result shows that, at 135 ${}^{{}^{\circ}}$C, all the modified mixtures would be able to achieve higher field densities than 95 %G_mm_ (requirement of the Superpave 5 mix design) with the regular field compaction effort. It is seen from Figure 9a,b again that the variability of compactability is higher at the lower compaction temperature.

### 4.2. Compaction Temperature Estimation

**Figure 10:**
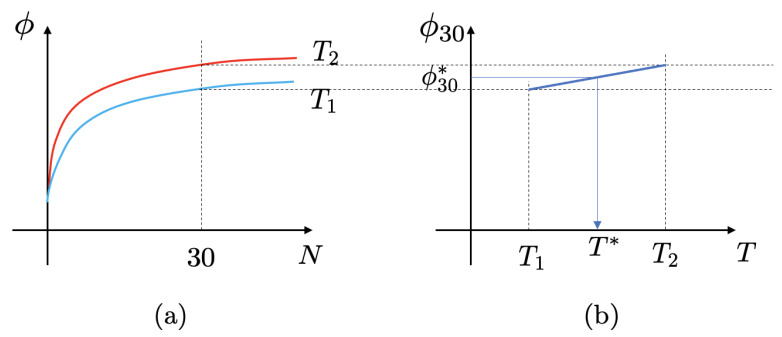
A schematic diagram for compaction temperature estimation: (**a**) Gyratory compaction curves at multiple temperatures, (**b**) compaction temperature interpolation.

A method is proposed for estimating the compaction temperature, which is based on the direct characterization of the compactability of asphalt mixtures by the gyratory compaction. A schematic diagram of this method is depicted in Figure 10. In this method, results of gyratory compaction curves at multiple (≥2) temperatures are required. Previous studies have shown that the field density is typically achieved at about 30 gyrations in the gyratory compaction [36,37]. Therefore, we use the density of mixtures at 30 gyrations, $\phi_{30}$, to estimate the field density level of the mixtures. For each mixture, the values of $\phi_{30}$ are obtained at the multiple temperatures, as shown in Figure 10a. Then, based on the $\phi_{30}$ results at the these temperatures, the compaction temperature $T^{*}$ is interpolated given the desired field density level $\phi_{30}^{*}$, as shown in Figure 10b.

In this study, gyratory compaction tests were performed at two temperatures: 95 ${}^{{}^{\circ}}$C and 135 ${}^{{}^{\circ}}$C. The $\phi_{30}$ results at the two temperatures are shown in Figure 7. As seen in Figure 10b, a model for the relationship between *T* and $\phi_{30}$ is needed for the interpolation of compaction temperature. Previous studies showed that the effect of temperature on compactability is nonlinear [50,51,52,53,54]. The change of compactability with temperature is more significantly at low temperatures than at high temperatures [52]. At extremely high temperatures (over 150 ${}^{{}^{\circ}}$C), temperature can even have a negative effect on compactability [50,51,54]. However, to the best knowledge of the authors, there is no generally accepted model for the relationship between compaction temperature and the compactability of asphalt mixtures. For simplicity and due to the limited number of data points, we employ a linear function as the first-order approximation to the nonlinear relationship between *T* and $\phi_{30}$.

(1) $$\phi_{30}=\alpha T+\beta ,$$

where α and β are model coefficients. It is important to note that this linear approximation model is acceptable only when compaction temperature is close to the experimental compaction temperatures, while it is expected that the linear approximation would become increasingly inaccurate as compaction temperature gets away from the experimental compaction temperatures. The linear model is applied to the four mixtures. The model coefficients are obtained and listed in Table 2.

**Figure 11:**
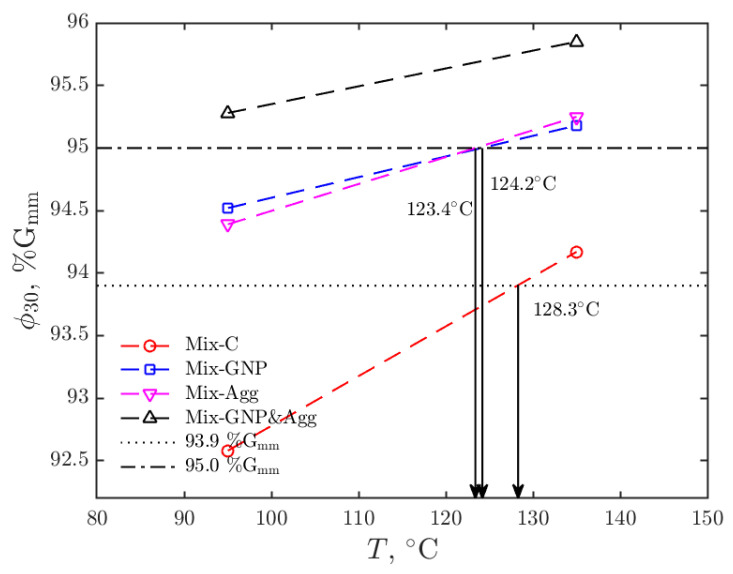
Estimation of the compaction temperatures by the linear model.

The linear approximations of the relationships between *T* and $\phi_{30}$ are shown in Figure 11 for the four mixtures. Again, it is seen that the compactability of Mix-C is the most temperature-sensitive, followed by Mix-Agg, Mix-GNP, and Mix-GNP&Agg.

Based on the obtained linear models between *T* and $\phi_{30}$, we can then estimate the compaction temperatures of the mixtures for achieving the desired field density level $\phi_{30}^{*}$. In this study, we consider two different $\phi_{30}^{*}$ levels: 93.9 %G_mm_ and 95 %G_mm_, which is the average field density of the control mixture (Mix-C) and the required field density level of the Superpave 5 mix design, respectively. As shown in Figure 11, for $\phi_{30}^{*}$ = 93.9 %G_mm_, the compaction temperature for Mix-C is interpolated as 128.3 ${}^{{}^{\circ}}$C. For the other mixtures, it is clear that the compaction temperatures are lower than 95 ${}^{{}^{\circ}}$C. However, their compaction temperatures cannot be accurately estimated due to the limitation of the linear model. For $\phi_{30}^{*}$ = 95 %G_mm_, the compaction temperatures for Mix-GNP and Mix-Agg are interpolated as 124.2 ${}^{{}^{\circ}}$C and 123.4 ${}^{{}^{\circ}}$C, respectively. The compaction temperature for Mix-C is clearly higher than 135 ${}^{{}^{\circ}}$C and the compaction temperature for Mix-GNP&Agg is clearly lower than 95 ${}^{{}^{\circ}}$C. However, only the estimation of the bounds can be made instead of the exact values, because of the limitation of the linear model. The estimated compaction temperatures for the four mixtures are listed in Table 3.

As shown in Figure 11, the modified mixtures can significantly reduce the compaction temperature of the control mixture. For both the two field density levels, Mix-GNP&Agg would need the lowest compaction temperature followed by Mix-GNP, Mix-Agg, and Mix-C. As shown in Table 3, for achieving the field density level of 93.9 %G_mm_, the compaction temperatures are lower than 95 ${}^{{}^{\circ}}$C for the modified mixtures, which means the compaction temperature can be reduced by more than 30% compared to the original compaction temperature (135 ${}^{{}^{\circ}}$C). For achieving the field density level of 95 %G_mm_, Mix-C need a compaction temperature higher than 135 ${}^{{}^{\circ}}$C, while the compaction temperatures are reduced to lower than 135 ${}^{{}^{\circ}}$C for the modified mixtures. In particular, the compaction temperature for the most compactable mixture, Mix-GNP&Agg, is reduced to even lower than 95 ${}^{{}^{\circ}}$C. These results show that the three methods can effectively reduce the compaction temperature of mixtures, with their effects comparable to the temperature reduction effect of WMA.

It is worthwhile to discuss the potential ways for improving the accuracy of compaction temperature estimation. One possible way is by increasing the number of experimental compaction temperatures, which would enable the characterization of the nonlinear relationship between *T* and $\phi_{30}$. Another way is by developing more realistic theoretical models for the *T*-$\phi_{30}$ relationship.

## 5. Conclusions and Recommendations

In this paper, two approaches for improving the compactability of asphalt mixtures were introduced. One is the addition of Graphite Nanoplatelets (GNPs), and the other is the optimization of aggregate packing. An experimental study was performed to estimate the effects of the two approaches and their combination on reducing compaction temperatures. A method for determining compaction temperature was proposed based on the gyratory compaction. The main conclusions of this research are summarized as follows.

1 GNP modification, aggregate packing optimizing, and their combination can significantly improve the compactability of mixtures and thus reduce the compaction temperatures.
2 The combination of GNP modification and aggregate packing optimization has the most significant temperature reduction effect, followed by GNP modification, and aggregate packing optimization.
3 The three methods can reduce the compaction temperature of the original mixture by more than 30%, which is comparable to the temperature reduction effect of WMA.
4 The proposed method for compaction temperature estimation is based on the direct characterization of the compactability of asphalt mixtures. It can take into account many affecting factors which were not possible to consider with the traditional viscosity-based method, for example, the properties of aggregates and the lubricating effect of binder. Thus, the method can potentially be used in practice for estimating the compaction temperatures for asphalt mixtures.

While the results presented in this proof-of-concept study are based on limited experimental data, it is reasonable to conclude that the proposed two methods (GNP modification and Aggregate packing optimization), and their combination, have good potential as practical methods for reducing the compaction temperatures of asphalt mixtures. It was also observed that the variability of the compactability of mixtures increased with the decrease in the compaction temperature, which implies that compaction temperature would influence the reliability of field compaction. Such an effect was not considered in the compaction temperature estimation in this study. It is recommended that future studies can consider the effect of compaction temperature on the reliability of field compaction. Moreover, it is seen from this study that one of the main difficulties for the compaction temperature estimation is the lack of knowledge on the relationship between the compaction temperature and the compactability of mixtures. To improve the accuracy of compaction temperature estimation, future research is suggested to explore the relationship between the compaction temperature and the compactability of mixtures, using a combination of experimental investigation and theoretical modeling.

## Figures and Tables

**Table 1:** Material information of the Mix-C.

Mix ID	NMAS (mm)	Binder PG	% AC	% Virgin AC	% RAP	FAA (%)	CAA1 (%)	CAA2 (%)
Mix-C	12.5	58S-28	5.6	4.2	30	42	99	93

Note: NMAS = nominal maximum aggregate sizes, PG = performance grade, AC = asphalt content, RAP = reclaimed asphalt pavement, FAA = fine aggregate angularity, CAA1 = coarse aggregate angularity of one fractured surface, CAA2 = coarse aggregate angularity of two fractured surfaces.

**Table 2:** Coefficients of the linear model between *T* and $\phi_{30}$.

Coefficients	Mix-C	Mix-GNP	Mix-Agg	Mix-GNP&Agg
α, %/${}^{{}^{\circ}}$C	10.42	4.32	5.63	3.74
β, %	71.97	85.97	83.24	87.89

**Table 3:** Estimation of compaction temperatures.

$\phi_{30}^{*}$, %G_mm_	Mix-C	Mix-GNP	Mix-Agg	Mix-GNP&Agg
93.9	128.3 ${}^{{}^{\circ}}$C	<95 ${}^{{}^{\circ}}$C ${}^{†}$	<95 ${}^{{}^{\circ}}$C ${}^{†}$	<95 ${}^{{}^{\circ}}$C ${}^{†}$
95.0	>135 ${}^{{}^{\circ}}$C ${}^{†}$	124.2 ${}^{{}^{\circ}}$C	123.4 ${}^{{}^{\circ}}$C	<95 ${}^{{}^{\circ}}$C ${}^{†}$

Note: † a bound is given for the compaction temperature, because the exact compaction temperature cannot be estimated due to the limitation of the linear approximation.

## Data Availability

Data are available from the corresponding authors by request.
